# A Bibliometric Analysis of the Scientific Research on Artisanal and Small-Scale Mining

**DOI:** 10.3390/ijerph19138156

**Published:** 2022-07-03

**Authors:** Fernando Morante-Carballo, Néstor Montalván-Burbano, Maribel Aguilar-Aguilar, Paúl Carrión-Mero

**Affiliations:** 1 Facultad de Ciencias Naturales y Matemáticas (FCNM), ESPOL Polytechnic University, Guayaquil 09015863, Ecuador; 2 Geo-Recursos y Aplicaciones (GIGA), ESPOL Polytechnic University, Guayaquil 09015863, Ecuador; 3 Department of Economy and Business, University of Almería, Carr. Sacramento s/n, La Cañada de San Urbano, 04120 Almeria, Spain; nmb218@inlumine.ual.es; 4 Centro de Investigaciones y Proyectos Aplicados a las Ciencias de la Tierra (CIPAT), ESPOL Polytechnic University, Guayaquil 09015863, Ecuador; pcarrion@espol.edu.ec; 5 Facultad de Ingeniería en Ciencias de la Tierra, Campus Gustavo Galindo, ESPOL Polytechnic University, Guayaquil 09015863, Ecuador

**Keywords:** mining, artisanal mining, small-scale mining, environment, bibliometric analysis

## Abstract

Mineral resource exploitation is one of the activities that contribute to economic growth and the development of society. Artisanal and small-scale mining (ASM) is one of these activities. Unfortunately, there is no clear consensus to define ASM. However, its importance is relevant in that it represents, in some cases, the only employment alternative for millions of people, although it also significantly impacts the environment. This work aims to investigate the scientific information related to ASM through a bibliometric analysis and, in addition, to define the new lines that are tending to this field. The study comprises three phases of work: (i) data collection, (ii) data processing and software selection, and (iii) data interpretation. The results reflect that the study on ASM developed intensively from 2010 to the present. In general terms, the research addressed focuses on four interrelated lines: (i) social conditioning factors of ASM, (ii) environmental impacts generated by ASM, (iii) mercury contamination and its implication on health and the environment, and (iv) ASM as a livelihood. The work also defines that geotourism in artisanal mining areas is a significant trend of the last decade, explicitly focusing on the conservation and use of the geological and mining heritage and, in addition, the promotion of sustainable development of ASM.

## 1. Introduction

Mining is a type of extractive activity considered to be one of the most important sources of metals and non-metals [1,2]. This activity is not always carried out by large-scale companies or industrial machinery; being called small-scale or artisanal mining. Small-scale mining (SSM) was first defined by the United Nations (UN) as: “Any single mining operation that has an annual raw material production of 50,000 metric tonnes or less, measured at the mine entrance” [3]. However, despite referring to the production magnitude or exploitation size, this concept differs at the level of countries and institutions. For example, in Brazil, the National Department of Mineral Research (DNPM) defines SSM as an operation that produces between 10,000 t/a (tonnes per year) and 100,000 t/a of ore [4]. On the other hand, in Ecuador, according to the mining law, the SSM exploits and processes up to 300 tons of ore per day (tpd) [5].

The SSM can be developed technically (conventional) or in a rudimentary way. When the operation of the SSM is conventional, it is characterised by being developed under a legal situation and the technical application of mechanised exploitation, as well as being processed with engineering criteria and feasibility studies that guarantee the results of mineral production [6]. On the other hand, when the operation is carried out through simple and rudimentary techniques to extract ore without conventional ecological and engineering principles, it is called artisanal mining (AM) [7]. Currently, no country has clear regulations defining activities classified as AM, and almost all policies only refer to the size of the operation [8]. Hilson [9] describes that artisanal mining exploitation involves “intense labour activity located in remote and isolated sites using rudimentary techniques, low technological knowledge, low degree of mechanization and low levels of environmental, health and safety awareness”. This term refers to the rudimentary type of exploitation, regardless of whether the mine is small or large [10].

**Figure 1:**
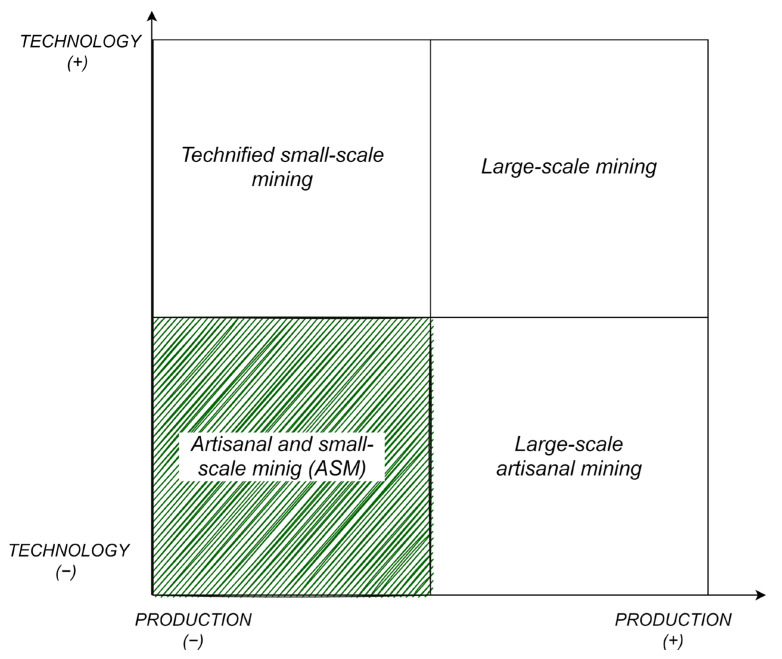
Schematic representation of ASM as a term that unites SSM and AM.

Artisanal mining and small-scale mining are used synonymously to refer to mining activity carried out by individuals or small groups with low technology or machinery [11]. Considering their close relationship, the legislations of developing countries refer to the term “artisanal and small-scale mining (ASM)” as “individuals, groups, families or mining cooperatives with minimal or no mechanization, often in the informal (illegal) sector of the market” [12] (Figure 1).

However, the definition of ASM is not uniform across many jurisdictions. Although there is still no internationally agreed upon definition of ASM, country-specific definitions reflect relevant situations and developments at the local level [13]. According to Seccatore et al. [7], “the term ASM is widely used to refer to those small or large operations that use rudimentary techniques to extract gold that operate legally or illegally and that are not on the radar of many companies mining companies, governments and international environmental agencies”. Various authors have studied and characterised this type of activity [12,14,15,16,17,18,19,20,21,22,23].

In general, ASM is an activity that exploits small deposits, has poor capital, lacks standards to ensure health and safety, is labour intensive, and has a significant environmental impact [14]. According to [24], millions of people worldwide are dedicated to primitive mineral extraction (ASM). Most ASM operators mine precious metals and stones [25]. Other mineable materials, such as minerals, include diamonds, columbite-tantalite, and bauxite [26,27,28].

Recent studies have focused on large and medium-scale mining effects, updating sustainable and environmentally responsible production techniques [29,30,31]. However, the effects produced by ASM are still a reality due to economic, legislative, and technological limitations [22,32,33]. Furthermore, ASM has witnessed a massive expansion worldwide, employing millions of people [14,34] and producing 15–20% of the world’s mineral production [7]. In addition, the areas where activities related to small-scale mining are located are studied, among other topics, from a geological point of view. In particular, in works oriented to the definition of the type of existing deposit (e.g., [35,36,37]), the characterization of the existing minerals of interest (e.g., [38,39,40]), and to the proposal of efficient exploitation alternatives (e.g., [41,42,43]).

Artisanal mining is driven by poverty, growing as an economic activity and adopted as a promising, and in many cases unique, alternative income [44]. However, ASM continues to develop without regulatory control in most developing countries, generating social and environmental problems in which crime, child labour, soil erosion, mercury contamination, and mining conflicts stand out [45]. The leading solution proposed by academics and professionals consists of improving ASM’s environmental, technical, and socioeconomic performance by implementing regulations that organize and formalize the sector, respecting miners’ rights [12,19,34].

Several literature review studies related to ASM mainly focus on systematic reviews of specific topics. Some examples are review studies about its relationship with poverty [24,46], agriculture [47], operator health [48], ecological problems [49,50], health risks [51,52], mercury contamination [6], mercury management and treatment [53], and water contamination [54], among others.

To date, no holistic analysis of ASM is recorded. This is possible with a bibliometric study that allows for knowing the structure and evolution of this field of research. Bibliometric analysis is a method that assesses the structure and trends of research in a specific body of literature [55,56,57,58,59,60], commonly used to categorize aspects of science as journals, institutions, universities, authors, and most contributing countries [61]. According to [62], this type of study is important for (i) obtaining a comprehensive overview of the subject under investigation, (ii) identifying knowledge gaps, (iii) defining novel lines in research, and (iv) positioning their contributions in the researched field. Bibliometric analysis can use two procedures: (i) analysis of scientific production, which leads to an evaluation of the impact of the field being investigated in the study and its scientific actors (authors, institutions, countries) [63,64]; and (ii) bibliometric mapping combined with clustering techniques that allow for evaluating of the cognitive structure and behaviour of the scientific field through the analysis of research fields, disciplines, and themes [65,66].

Based on the above, and considering the conflict (similarity and variation of definitions between SSM and AM), the following research question arises: How should we organize information to carry out a comprehensive analysis of the evolution and trends of the scientific production of the SSM and AM?

In this study, the term ASM is considered as a holistic concept that integrates SSM and AM as synonyms of low-production mining activity, characterised by the low-quality technology used and intensive labour. For this reason, the objective of this study is to analyse the existing literature base related to ASM through bibliometric methods that allow for the definition of the main areas being investigated, patterns, trends, and the proposal of new lines of research.

The article consists of six main sections: the introduction (Section 1), which includes a review of scientific literature related to ASM in the world; materials and methods (Section 2), which describes the procedure used in this study; results (Section 3), in which the results obtained from the analysis and processing of the database are presented; discussion (Section 4), which lies in exposing the importance of the study and the determination of future lines of research; the conclusions (Section 5), which include the limitations of the study; and finally, the references used which support this research.

## 2. Materials and Methods

Bibliometric research, a meta-analytic literature research tool, was conducted in this study [57,67]. This type of study is about analyzing (mapping) the structure, evolution, and research trends of a specific database [55,56,58,59,60,68] through parameters such as authorship, citations, keywords, journal, and affiliations [61,69].

**Figure 2:**
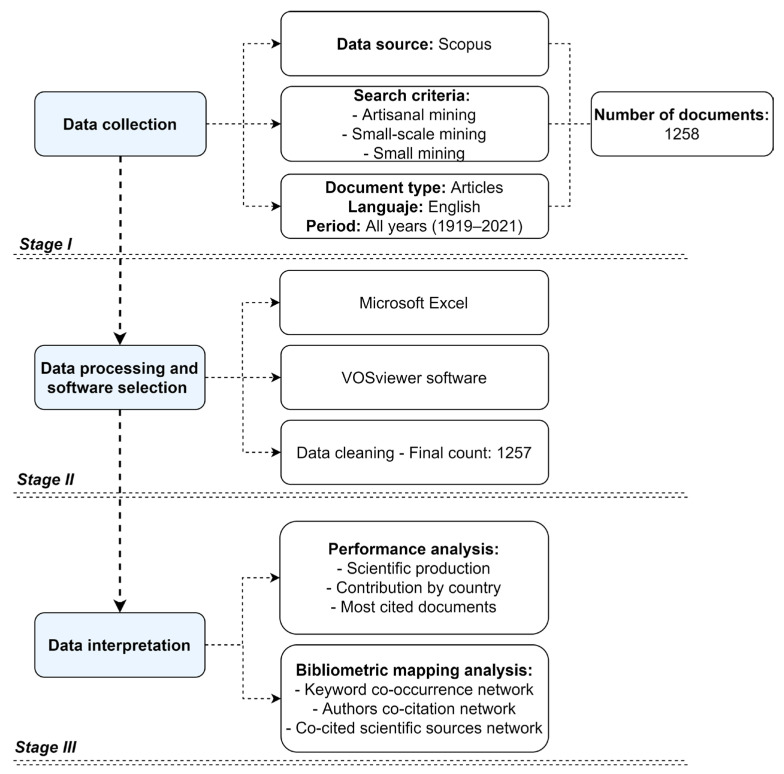
General methodological scheme of the study.

For the bibliometric analysis of a specific field of research, it is necessary to use bibliometric maps [70,71], which can be viewed in different software (e.g., Bibexcel, CitNetExplorer, CiteSpace, CoPalRed, HistCite, Net-work Workbench Tool, SciMAT, Sci2Tool, VantagePoint, and VOSviewer). This study used the VOSviewer software [65] to build bibliometric networks in order to facilitate the analysis of the intellectual structure using various parameters obtained from scientific publications [72]. The research contemplates a systematic process distributed in three phases (Figure 2): (i) data collection, (ii) data processing and software selection, and (iii) data interpretation.

### 2.1. Data Collection

Most of the research literature on small-scale mining is closely related to artisanal mining [34,73,74]. Furthermore, scientific contributions on artisanal and small-scale mining (ASM) generally expose case studies, mainly in developing countries, in which small-scale mining is a term frequently used to refer to artisanal mining activity [25,75]. Therefore, considering this relationship, the following search terms are considered in this study: (i) artisanal mining, (ii) small-scale mining, and (iii) small mining. The selected terms will allow for the obtaining of a complete literary body on the subject for its later bibliometric analysis.

Quality databases with accurate and consistent information are essential [76,77]. Therefore, the Scopus database was selected for the search, as it is considered one of the central databases with great coverage, facilitating the study and comparison of different scientific fields [78,79,80,81,82,83]. In addition to its comprehensive coverage and ease in the tools provided for bibliometric analysis, in this specific study (artisanal and small-scale mining), we considered the main reason for the extensive coverage of Scopus in terms of scientific production related to geosciences [84,85,86].

Scopus constitutes an indexed and well-organised database of scientific production, with tools that allow the export of metadata [63,80,87]. In addition, it provides a series of data on scientific publications such as authors, institutions, countries, number of citations, and research areas [78,80,88,89]. An important aspect to consider in selecting the database is that the growth in the coverage of journals from Latin America and the Caribbean indexed within the Scopus database [90,91] strengthens the analysis carried out in different areas.

The search was carried out on 8 November 2021, using the terms previously defined in the titles, abstracts, and keywords of the different existing publications in Scopus. The initial search equation used was: ((TITLE-ABS-KEY (“artisanal mining”) OR TITLE-ABS-KEY (“small scale mining”) OR TI-TLE-ABS-KEY (“small mining”))), with a result of 1665 documents. Subsequently, the database was delimited through inclusion and exclusion criteria according to the analysis to be carried out. As a first criterion, it was considered appropriate to exclude the year 2022 and carry out the study with documents published up to the present (search date). Subsequently, the number of documents was limited to articles, since the results obtained from the initial search equation yielded more than 75% of documents as articles. Finally, considering that the English language is the most frequent in scientific publications [92], the initial search of the investigated area indicated that more than 90% of documents are written in English; the study was limited to documents in that language. The final database represents 1258 documents, which will be the basis for processing phase two of the study.

### 2.2. Data Processing and Software Selection

The data processing and software selection phase begins with extracting data from the Scopus database through a Microsoft Excel spreadsheet. The software uses data analysis and error elimination [93,94,95] and evaluated the investigated area’s scientific production [96]. Specifically, the downloaded database contains authors, titles, keywords, years, number of citations, and abstracts. Then, a cleaning and error elimination process is carried out [97,98], eliminating repeated and incomplete data for this research, obtaining 1257 documents to analyse.

With the adjusted database, we construct two-dimensional bibliometric networks, which define the research structure of the field being studied using the VOSviewer software (Version 1.6.17) [65]. The software is freely available and is used as the primary tool for constructing detailed bibliometric maps through simple graphs [70,99,100]. This software is used in different scientific areas such as medicine [101,102,103,104], management [105,106,107,108,109], natural and cultural resources [110,111,112], and geosciences [68,113,114,115,116], among others.

### 2.3. Data Interpretation

The investigated field analysed the results through (i) performance analysis and (ii) scientific mapping [117]. The first analysis makes it possible to determine the evolution of scientific production and its impact by evaluating parameters such as authors, year, affiliations, journals, and countries [118,119,120]. The subsequent analysis (scientific mapping) allows for the definition of different relationships between the analysed variables, obtaining information at the micro-level (co-occurrence of author keywords), meso-level (co-citation of authors) and macro-level (journal co-citation) [94,121]. Specifically, the objective of the analysed approaches was to identify the main areas of research on ASM for the definition of new lines based mainly on innovative, sustainable, and affordable research.

## 3. Results

### 3.1. Performance Analysis

#### 3.1.1. Scientific Production Analysis

Research studies related to artisanal and small-scale mining (ASM) began in 1919, with the study of Wormleighton [122], which marked the interest in this type of research on sewage and drainage works in a mining district. However, the first five decades (until 1979) of research in this field are scarce, with eight articles representing 0.63% of the total scientific production of ASM. Due to these reasons, excluding these years from the production analysis is considered pertinent.

**Figure 3:**
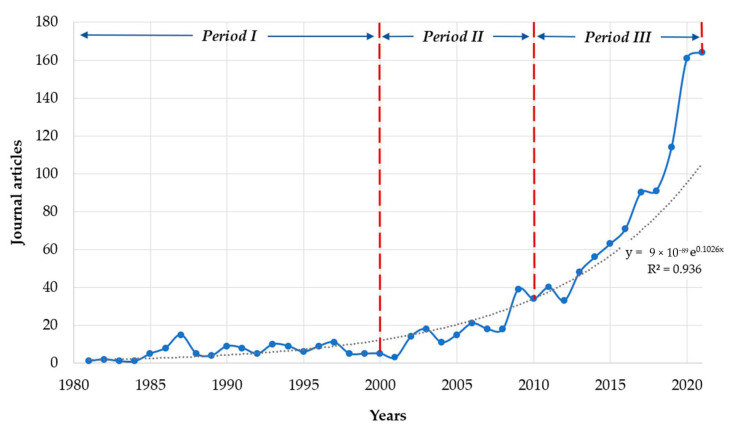
The behavior of ASM scientific research over time (1981–2021).

This analysis is divided into three periods distributed by decades: period I (from 1981 to 2000), period II (from 2001 to 2010), and period III (from 2011 to 2021) (Figure 3). For period I, two decades are grouped (1981–2000) due to the low number of published articles.

*Period I (1981*–*2000):* This research period of ASM in the world begins with 124 scientific articles, in which a similar production trend can be observed every five years (Figure 3). It is essential to highlight that in 1987 the highest production was obtained within the analysed period (Figure 3), with 15 published articles. In general, this first period marks the beginning of ASM research. The primary study topics focus on the contribution of small-scale mining to world mineral production [123], as well as its contribution to the socioeconomic development of developing countries [124]. Likewise, case studies of small-scale mining [125,126,127,128,129], the role of the government in promoting small-scale mining [130], and the need for government policies [131,132] are presented.

Within this research period, the authors also expose the importance and characteristics of small-scale mining [133], as well as the primary technical considerations that reduce the human and environmental risk [134], despite its limitations [135]. Likewise, it is possible to observe studies focused on the pollution problems of small-scale mining [136,137] (e.g., in water [138], soil [139], and environment [140,141]), seismicity inductions [142,143], and mining-associated diseases [144].

*Period II (2001*–*2010):* This decade is characterised by significant growth in research, with a total of 191 articles representing 15.15% of the ASM research field. In 2009 there was a peak in research with 39 publications (Figure 3). Ranging from 2001 to 2010, ASM research is related to mining environmental management [145,146,147,148] and the need for mining legislation [34,149,150,151,152] that will solve environmental pollution problems [42,153,154,155,156,157,158]. During this period, studies on illegal mining are also visible [159,160,161,162], which generate land-use conflicts due to small and large-scale mining [163,164]. On the other hand, it is essential to highlight the increase in the scientific production of gold ASM, in which the scarce legislation [147,152,154,165,166], problems of health in people [167,168,169], and the inclusion of women in this type of activity [170] are emphasised.

*Period III (2011*–*2021)*: Finally, the third period analysed is characterised by an exponential growth in scientific production related to ASM, with a total of 911 articles representing 74.21% of the total documents analysed (Figure 3). The average annual production exceeds 80 articles, with a peak in 2020 (161 articles) and 2021 (164 articles) investigated, defining ASM as a booming research field. As mentioned in previous periods, this field of research is generally related to lines such as pollution [49,171,172,173,174,175,176,177], agriculture problems caused by ASM [178,179,180,181], the association of ASM with poverty [182,183,184], mining conflicts [185,186], informal/illegal ASM [187,188,189], and the influence of ASM on water quality [190,191], among others. However, this period is characterised by an intensive growth in the scientific contribution to solving mining conflict problems through ASM formalization strategies [45,192,193,194,195,196,197,198], in addition to contributing to research focused on strategies for reducing environmental pollution [199,200] and health risk mitigation [201].

#### 3.1.2. Regional and Country Contribution

**Figure 4:**
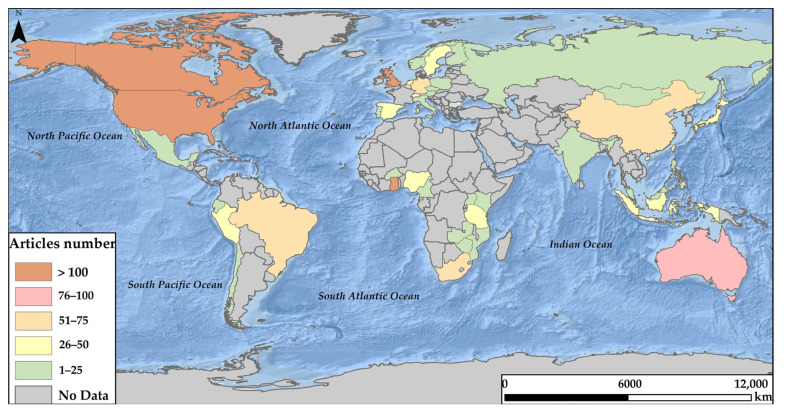
Contribution of studies related to ASM by country.

**Figure 5:**
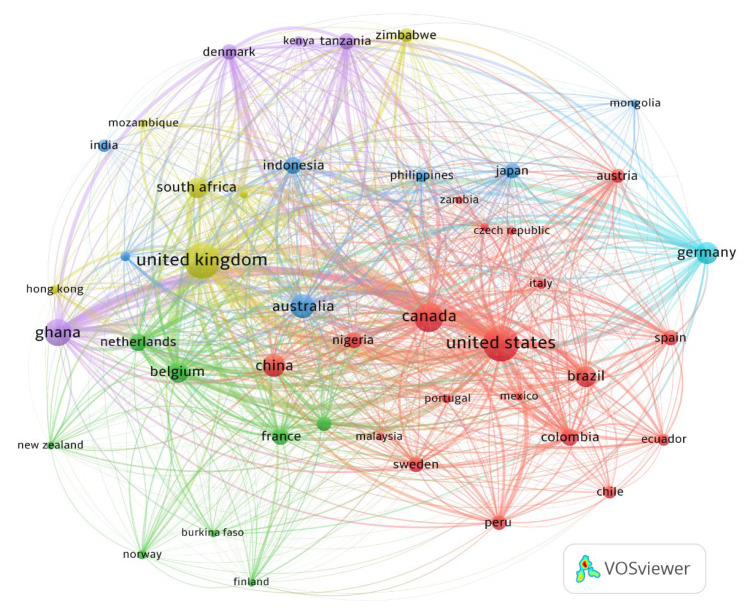
Country contribution bibliometric map in ASM. The nodes’ size varies depending on the number of documents per country, related through links in which their collaboration is reflected.

According to the authors’ different affiliations, the contribution by country indicates that, worldwide, 46 countries contribute to research related to ASM (Figure 4). In general, four countries stand out due to their high scientific production: the United States (210 articles), United Kingdom (209 articles), Canada (133 articles), and Ghana (109 articles) (Figure 5). In addition, these countries are characterised by a high number of citations compared to the other contributing nations, with the United Kingdom standing out as the most cited country worldwide on topics related to ASM (94,929 citations) (Table 1).

According to the affiliation obtained, it is essential to note that the top 10 countries that contributed the most in the field can be differentiated (Table 1), highlighting the participation of developed countries such as the United States, United Kingdom, and Canada, leaders in ASM research throughout the world.

The behaviour of collaboration between countries, based on affiliation data, indicates that the United States, Canada, Australia, Germany, Austria, and Spain are the countries with the most significant collaboration (each one collaborates with 45 different countries). The United States, the country with the highest production, contributes to 45 countries, of which Canada, Ghana, and Germany stand out. When analyzing the United States production, the studies focus on issues related to the impact that ASM generates on the environment [49,156,157,202,203], health implications [168,171,173,204,205,206], the effects of AMS on socioeconomic factors [24,184,207], and the inclusion of women in jobs related to this type of activity [23,170,208]. Strengthening its studies of problems associated with ASM, the United States also generated contributions in the areas focused on the need for ASM regulations [195,209,210,211], as well as ASM risk and contamination mitigation alternatives [201,212,213,214].

Although the United States is the country with the highest scientific production, the United Kingdom, with only one less article, far exceeds the number of citations in its works. These studies include the socioeconomic impacts of ASM in developing countries and strategies focused on the sector’s sustainability [9], environmental problems of small-scale gold mining [42], poverty-driven informal artisanal gold mining [73], and ASM reforms [215]. This analysis also includes the study of the dependence on mercury as an agent of poverty in artisanal gold mining [216] and the pollution generated in these communities [217]. Studies on strategies to eradicate illegal artisanal mining are also included [162].

Canada, occupying third place in the contribution of ASM articles, makes contributions focused on African or South American countries. The investigations are related to the current use of mercury in ASM [7] and the proposal of actions focused on the reduction of these types of emissions [218], as well as the responsibility of miners, governments, and organizations in the search for solutions to pollution problems [41,219,220]. There are also studies related to the role of ASM formalization in Africa [34].

#### 3.1.3. Journal Performance

The analysis included 468 journals in which 1257 scientific articles were published (database analysed) related to ASM. Table 2 shows the top 10 of the most outstanding journals, with 401 articles representing 31.9% of the total.

*Resources Policy* is the leading journal in scientific publications in the analysed field with 116 articles representing 9.2% of the total. This journal is the most cited worldwide, with 2912 citations. The top five studies with the highest citations (Banchirigah [162], Hilson [221], Siegel & Veiga [34], (Mohammed Banchirigah [215], and y Geenen [193]) focus on formalization and poverty related to ASM in Africa. Based on its citations (163), the most relevant study was developed by Banchirigah [162] in Ghana. The study argues for the need to eradicate illegal mining through formalization, work alternatives, and government and military intervention. On the other hand, the journal *Science of the Total Environment*, occupying fifth place in the production of ASM, represents the second most cited journal (1492 citations). The two most cited articles correspond to the one carried out by Hylander and Goodsite [157] (191 citations) and de Cordy et al. [41] (162 citations), which discuss mercury contamination from ASM and the costs involved in remediating the environment.

#### 3.1.4. Frequently Cited Documents

Citation analysis exposes a given article’s influence by the citation it receives in another articles [222]. The scientific production for ASM globally (1257 articles) presents 20,579 citations. Table 3 presents the top 10 of the most cited documents with 1776 citations, representing 8.63% of the total. The established ranking is characterised by documents published in 2005.

The study by Bebbington et al. [223] is the most cited article (292 citations), with intervention of authors from the United Kingdom, the United States, Ecuador, and Peru. In his study, reference is made to the influence of social movements against mining investment in Latin America. Mainly two case studies are exposed (Ecuador and Peru), in which it is evident how social activities can significantly modify the form and effects of the extractive industry.

Second place is occupied by Xiao et al. [173], with the presence of authors from China and the United States. The research analyses soil contamination from artisanal gold mining in China and its implications for human health and environmental wellbeing by assessing heavy metal levels in soil and plants. Likewise, within its objectives, the identification of plants that promote the phytoremediation of the area is addressed.

Finally, the third most cited article related to ASM is the work developed by Hilson and Potter [73], authors from the United Kingdom. Their scientific contribution focuses on analysing Ghana’s National Structural Adjustment Program (SAP) as a driver in the growth of informal artisanal gold mining driven by poverty.

### 3.2. Intellectual Structure Analysis

#### 3.2.1. Co-Occurrence Author Keyword Network

The co-occurrence analysis of author words allows for the formation of connections and the building of a domain structure based on keywords [225]. The analysis included a process of cleaning and filtering the information, obtaining 90 keywords. Table 4 shows the top 15 words with the highest occurrence in the area studied, highlighting “artisanal and small-scale mining”, “mercury”, and “mining” as the top three most frequent keywords in ASM studies.

**Figure 6:**
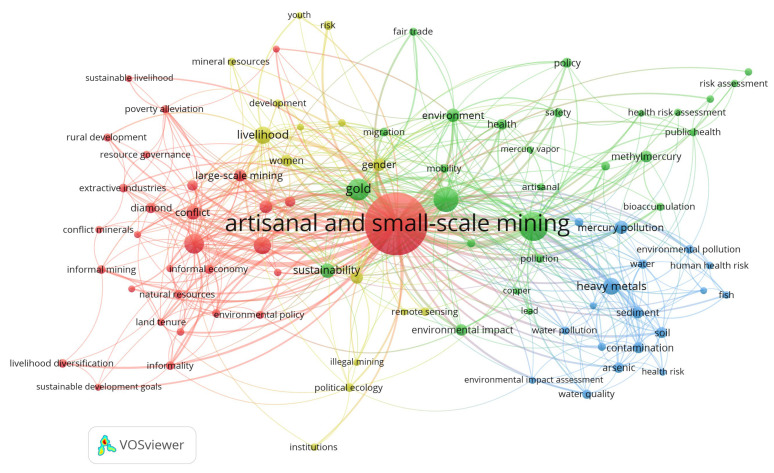
Author keyword co-occurrence bibliometric map in ASM.

The bibliometric map obtained grouped the 90 keywords into nodes of different colours grouped into four clusters that represent the main research areas of ASM (Figure 6). The nodes’ size varies depending on the number of occurrences of each keyword, and they are related through links in which the thickness represents a better relationship.

##### Cluster 1 (Red Colour): Social Conditioning Factors of the ASM

The social conditioning factors of ASM represent one of the research areas aimed at understanding how poverty drives the development of this type of activity as a subsistence alternative, which entails informality [189], conflict [185,186], child labour [226], and women’s labour [227]. Likewise, the link between mining and agricultural activity in rural areas with low economic resources is exposed as the primary source of subsistence for people [27,180,228]. Considering this type of problem, it is evident how formalization represents a considerable challenge [186] and is regarded as a tool that allows for regulating, controlling, and effectively supporting ASM operators [34,45,197,209,229]. However, several case studies show that formalization in various countries aggravates mining conflicts, informality, poverty, illegality, and state control [193,230,231,232]; entrenching poverty without achieving sustainable development [233].

Given this situation, research developed to establish strategies in ASM that allow for achieving sustainable development [234] through an analysis of social, political, economic, environmental, and health aspects [235,236,237]. Some examples of this type of action are: (i) the implementation of design thinking and multi-criteria decision analysis of ASM [238], (ii) national minerals policies and stakeholder participation in planning decisions [239], (iii) collaboration between the LSM and ASM, for the benefit of the communities [240], and (iv) integration of scientific and local knowledge in the planning of the remediation of contamination by ASM [214,241].

##### Cluster 2 (Blue Colour): ASM Environmental Impacts

Artisanal and small-scale gold mining (ASSGM) is the most developed activity in ASM. In this area of research, significant production of environmental and health impacts caused by ASSGM is evident [156,218,219], and limited studies are addressing the effects on the health and environmental impacts of artisanal sandstone mining [242] and diamond mining [26,234,243].

The investigations are most frequently related to pollution generated in the soil [244,245,246,247,248], water [249,250,251], and crops or trees [158,252], which directly influence the health and wellbeing of humans. Faced with this problem, finding innovative research to eliminate, replace, or reduce environmental pollution in mineral processing is standard. Some examples are the cyanide phytoremediation by water hyacinths (Eichhornia crassipes) in the cyanide effluents treatment in small-scale gold mining [253], hyperaccumulation of zinc by Corydalis davidii in Zn-polluted soils [254], Erato polymnioides as a phytoremediation plant for soils contaminated with Pb, Zn, Cu, and Cd [255], and Heliconia psittacorum in remediating soils and water polluted with heavy metals [256].

##### Cluster 3 (Green Colour): Mercury Contamination and Its Implication on Health and the Environment

Mercury is a heavy, liquid metal frequently used in artisanal gold mining. This cluster reflects a marked trend of studies focused on the health and environmental effects of mercury or methylmercury contamination in soil, sediments, and water [257,258,259]. This type of contamination generated significant research on health problems associated with direct or indirect exposure of humans to mercury due to mining activities [167,260,261,262,263], as well as studies evaluating the risk posed to human health by ingestion of heavy metals that are present in the water and plants [176,264,265,266].

Given the implications of mercury on the environment and health, the reason for the emergence of research that highlights the importance of cooperation between government, regional, and local organisations to improve mineral extraction and processing processes through legalisation, financial support, technological innovation, and training [9,212,267,268], as well as studies focused on reducing pollution to ensure human and environmental health [202,269,270], is evident. These include analyses that seek to minimise the use of mercury through price increases [219], laws (agreements) that prohibit its use in mining [269,271,272,273], promotion of appropriate technology [154,274], and training on improved technologies for gold extraction [275] (e.g., use of cassava to leach gold [276]). Finally, it is essential to highlight how local participation in decision making [277] and indigenous participation due to their ecological knowledge [278] are alternatives to achieving sustainability in ASM mineral processing.

##### Cluster 4 (Yellow Colour): ASM as Livelihood

In this cluster, the most frequent studies are those related to ASM as a subsistence activity in rural communities with limited resources. Within her research, the women’s role in ASM as a means of subsistence due to poverty is emphasised [227,279,280], as well as the need for policies that improve the economic wellbeing of people who depend on ASM regardless of gender [229,281]. On the other hand, considering that several countries chose to ban this type of mining, there is also research related to alternative livelihood strategies for miners who were displaced from their activity [282,283]. Some examples of these strategies are promoting agriculture as an alternative economic source [179,284] or complementary [178], and promoting government support in ASM through regulations that allow regulating activity [194].

#### 3.2.2. Co-Citation Network of Cited Authors

**Figure 7:**
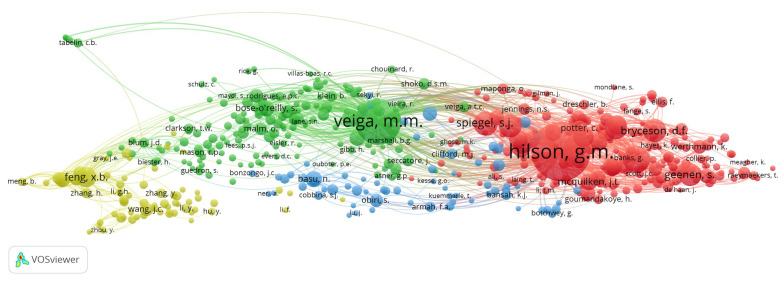
Co-citation network of authors in ASM.

The analysis carried out allowed for the identification of co-cited authors and authors that make up the scientific base of the area studied [285]. This type of analysis proposes that two authors share the same area of research if their documents are cited jointly by one or more documents [286,287,288]. The author co-citation network (Figure 7), built in the VOSviewer software, groups 512 authors (nodes) into six clusters representing similarities in the topics investigated with more than twenty co-citations.

Cluster 1 (red colour), “ASM and implications in society”, comprises 206 authors, including Hilson, G.M. (2212), Maconachie, R. (456); Spiegel, S.J. (363); Bryceson, D.F. (353); y Banchirigah, S.M. (337) due to its high number of co-citations. This group of researchers carried out studies within ASM that include: (i) positive and negative effects of artisanal mining formalization [194,197,198,215,289], (ii) ASM and agriculture as a means of subsistence [47,180,284,290,291], and (iii) analysis of alternatives that improve mineral extraction or processing systems [269,292,293,294].

Within cluster 2 (green colour), “consequences and challenges of Mercury in ASM”, the researchers Veiga, M.M.; Beinhoff, C.; Bose-O’reilly, S.; Telmer, K.H.; and y Drasch, G. represent the top five co-cited authors, in a cluster with a total of 166 authors. This research includes studies of mercury contamination in gold mining areas [41,295,296], evaluation of risks to human health due to exposure to mercury by operators, women, and children [167,295,297,298], and strategies to reduce this type of contamination based on the modernization of mineral processing in obtaining gold [148,199,219,299,300,301,302].

Cluster 3 (blue colour), “Implications of ASM in health”, composed of 73 authors, in which Basu, N.; Pardie, S.; Obiri, S.; Aryee, B.N.A.; and Amankwah, R.K. are the most coveted authors. This cluster mainly includes studies of risk to human health due to exposure to mercury [48,303], environmental impacts of ASM [49], consumption of contaminated food or water [304], or multiple heavy metals [305]. Likewise, the authors expose an interest in providing strategies to reduce pollution produced by ASM, mainly due to the use of mercury [155,216,217,234,242,306].

Finally, cluster 4 (yellow colour) with 67 authors, called “Effects of artisanal mercury extraction”, leads to the top five most co-cited authors, represented by Feng, X.B.; Qiu, G.L.; Li, P.; Wang, J.C.; and Wang, S.F. This group of authors dedicate their studies to topics related to Hg contamination in the air [307], water [308], sediments, soil, or crops [309,310,311,312] in mercury mining areas, mainly in China. They also analyse the risk posed to miners and people in mining areas when exposed to Hg or methylmercury [313,314,315].

#### 3.2.3. Journal Co-Citation Network

The analysis considers the similarity between a group of journals based on the citations received when two or more journals are cited jointly by several related documents [316]. The objective of this analysis is based on understanding the structures of the academic areas.

**Figure 8:**
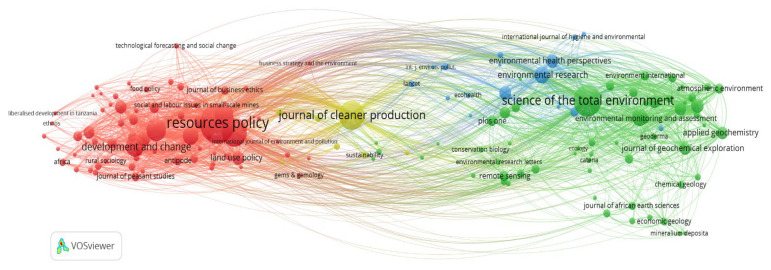
Journal co-citation network in ASM.

Figure 8 shows the co-citation network of 152 journals (nodes) with more than 20 citations, grouped into four different clusters (differentiated by colours) and their other connections.

Cluster 1 (red colour), “Management, Policy and Development”, contains 70 journals representing 8757 citations. In this group, the journals *Resources Policy* (1799 citations, United Kingdom), *Extractive Industries and Society* (939 citations, The Netherlands), *World Development* (578 citations, United Kingdom), *Natural Resources Forum* (526 citations, United Kingdom), and *Development and Change* (502 citations, UK) are shown as the top five of the most talked-about magazines. The studies within this cluster comprise analyses of ASM’s political, economic, environmental, and social aspects in different parts of the world.

Cluster 2 (green colour), “Environmental Science and Pollution”, with 58 journals and 5675 citations, mainly exposes studies associated with the environmental contamination of ASM and its human implications. In this group are journals such as *Science of the Total Environment* (1170 citations, The Netherlands), *Environmental Science & Technology* (365 citations, United States), *Environmental Pollution* (245 citations, United Kingdom), *Chemosphere* (205 citations, United Kingdom), and *Water, Air and Soil Pollution* (205 citations, The Netherlands), among others.

Cluster 3 (blue), “Environmental Science and Health”, has 16 journals and 1327 citations. The journals with the highest citations include *Environmental Research* (322 citations, United States), *Environmental Health Perspectives* (230 citations, United States), *International Journal of Environmental Research and Public Health* (230 citations, Switzerland), *Minerals Engineering* (101 citations, United Kingdom), and *International Journal of Occupational and Environmental Health* (68 citations, UK). Within this cluster, the primary studies focus on evaluating the impact of ASM on human health due to direct or indirect exposure to heavy metals.

Cluster 4 (yellow colour), “Renewable Energy, Sustainability and the Environment”, consists of 8 journals with 1305 citations. These journals include research papers focused on mineral extraction and processing sustainability in ASM. The top five most-cited journals are *Journal of Cleaner Production* (1000 citations, UK), *Environmental Science & Policy* (65 citations, The Netherlands), *Ecological Economics* (57 citations, The Netherlands), *Sustainability* (57 citations, Switzerland), and *Journal of Sustainable Mining* (54 citations, Poland).

## 4. Discussion

The systematic process applied in this study made it possible to identify the intellectual structure of artisanal and small-scale mining (ASM) in the world. Considering the performance analysis carried out, it is apparent that the scientific production of ASM began in 1919, being until 1980 a scarce production (eight articles). Furthermore, the range of years analysed (distributed in three periods) indicates that the research remained relatively constant since 1980 (periods I and II). However, as of 2010 (period III), ASM research increased exponentially worldwide, representing 74.21% of the articles produced (Figure 3). This marked difference in scientific production could refer to the artisanal mining boom that the world experienced in the last decade, mainly due to the increase in poverty within rural areas. The rise of ASM, characterised by extraction and processing techniques without technical and environmental considerations, clearly represents a risk to humanity and the environment. This is why the increase mentioned above in scientific production focuses its studies on ASM contamination [173,174], mining conflicts [185,186], illegal ASM [187,188,189], as well as strategies to solve these types of problems [196,197,200,201].

On the other hand, when analyzing scientific production by country, the United States, the United Kingdom, and Canada represent the most significant contributions to research related to ASM (Table 1). Of these countries, the United Kingdom is characterised by its high number of citations (Table 1) and its extensive collaboration (greater than 70%) in studies carried out in African countries (e.g., Ghana and Tanzania). Likewise, this country occupies the number one position with the *Resource Policy* magazine, contributing the highest number of ASM publications (116 articles) (Table 2). On the other hand, the United States and Canada collaborate in studies mainly in South American countries such as Brazil, Peru, and Colombia, and Africa, mainly in Ghana.

Considering the analysis of the intellectual structure through three scientific maps, the study of the co-occurrence of author keywords (Figure 6) made it possible to define, through clusters, four research areas of ASM. Within these areas, “Social conditioning factors of the ASM” and “Mercury contamination and its implication in health and environment” are the most studied topics (e.g., [34,192,209,218,240,252]). On the other hand, it is essential to highlight that cluster 2 (“ASM environmental impacts”) and cluster 3 (“Mercury contamination and its implication in health and environment”) are strongly related (Figure 6), with studies focused on the impacts of ASM on the environment (e.g., [249,251,258,309]) and health (e.g., [261,263,265]). However, considering a specific orientation and significant scientific production related to mercury, the results reflect the study of mercury as a particular area in this analysis.

Cluster 4 (ASM as livelihood) is an ASM area with relatively less scientific production, strongly related to cluster 1. The objective of ASM as a livelihood area includes research in which ASM is analysed as a means of subsistence and the search for strategies to propose alternative or complementary activities that benefit the living conditions of people who depend economically on this type of activity (e.g., [179,194,280]).

To complement the analysis of the co-occurrence of keywords, the co-citation analysis of authors was carried out, which allowed for the identifying of the relationships between different authors in the references of the research works carried out ion ASM. The results obtained reflect the existence of 512 authors grouped into four clusters, representing the author’s areas or lines of research (Figure 7). These areas are very well defined in specific topics; however, they are all within a large area called “Effects of ASM and mitigation measures”. Of the clusters obtained, clusters 2 and 3 are firmly related, presenting studies that address similar issues regarding the use and effects of mercury in ASM [216,219,297,303]. On the other hand, it is important to highlight an area aimed at research related to the artisanal extraction of mercury (Cluster 4), in which authors such as Feng, X.B.; Qiu, G.L.; Li, P.; Wang, J.C.; and Wang, S.F. carried out works that include the contamination generated by mercury mines in the soil, water, and air [307,308,309], as well as the risk it represents for human health [310,313].

Finally, the co-citation analysis of journals was carried out to understand the different academic areas in which ASM studies are published. The results show us four main academic areas (clusters) (Figure 8), defined based on the research topics. For example, in the cluster with the highest number of co-cited journals (cluster 1), the journals *Resources Policy* and *Extractive Industries* stand out with the highest number of co-citations in works oriented to ASM’s political, economic, social, and environmental aspects. Likewise, it is essential to highlight that clusters 2 and 3 show related academic areas in which the journals publish research topics on environmental pollution of ASM and its health risks. In these clusters, the journals with the highest number of co-citations correspond to *Science of the Total Environment* (cluster 2) and *Environmental Research* (cluster 3), which add up to 1170 and 322 citations, respectively.

On the other hand, the connection offered by cluster 4 (Renewable Energy, Sustainability, and the Environment) with the other clusters is visible. Being in the centre of the clusters obtained (Figure 8), despite its limited number of journals (eight), its high number of co-citations (1305) highlights the importance of its research topics focused on the socio-environmental aspects of ASM, with the *Journal of Cleaner Production* as the most prominent journal.

Specifically, ASM research exposes excellent studies that identify the causes and effects of the leading social, economic, and environmental problems that compromise environmental and human wellbeing in the short, medium, and long term (e.g., [9,34,73,157,173,218,223,317]). These studies lay the groundwork for issues that must be mitigated and eliminated. The analysed database reflects that, over time, studies developed that focus on solutions to problems generated by ASM (e.g., [238,239,241,253,256,268,278,294,318,319]). However, despite the worldwide importance and impact of research aimed at ASM solutions, it is still scarce (less than 20% of the analysed database). For this reason, the possibility arises that the different authors in ASM strengthen this type of study to the point that in the best of cases, it is considered one of the top research areas in ASM.

The analysis made it possible to evaluate the evolution and trends of research in ASM and propose strengthening innovative studies regarding ASM’s environmental, social, legal, and economical solutions. Therefore, this type of research can be included by the representative authors and journals of ASM as a new booming field that represents sustainable solutions for the effects produced by this type of mining activity.

## 5. Conclusions

The bibliometric analysis allowed us to evaluate the structure of ASM research field within the last four decades. Within the performance analysis, the results obtained show a scientific production with exponential growth in ASM research, with the collaboration of 46 countries, highlighting the United States, United Kingdom, and Canada as the countries with the highest scientific production that address ASM research in mainly Latin American and African countries, respectively. Furthermore, the works are the products of 512 authors published in 468 journals, qualifying ASM as a booming research field.

By analysing the co-occurrence of keywords, four areas of research in ASM were defined: (i) social conditioning factors of ASM, (ii) environmental impacts generated by ASM, (iii) mercury contamination and its implication on health and the environment, and (iv) ASM as a livelihood. Within these areas, a clear trend of studies related to the implications of ASM from the political, social, economic, and environmental points of view is apparent. On the other hand, it is essential to highlight the effects of mercury on the environment and health as topics on the rise, mainly in health risk assessment and strategies that minimise the impact of mercury on ASM. However, studies aimed at finding solutions in ASM to date are scarce and need to be strengthened.

Despite limiting the study to only one database (Scopus) and considering only one type of document (articles) in the English language, the proposed research establishes a global analysis of the ASM study. This analysis can serve as a reference for future researchers in the field for the most researched topics, authors, and outstanding journals; and raise the possibility of forming collaborative networks inside and outside your country.

## Figures and Tables

**Table 1:** Top 10 countries by the number of documents.

Ranking	Country	Region	Documents	Citations
1	United States	América	210	3989
2	United Kingdom	Europa	209	6440
3	Canada	América	133	2891
4	Ghana	África	109	1792
5	Australia	Oceanía	83	1076
6	China	Asia	71	1508
7	Germany	Europa	67	1209
8	Brazil	América	63	931
9	South Africa	África	57	394
10	Belgium	Europa	56	1307

**Table 2:** Top 10 journals with the highest number of publications.

Ranking	Journal	Country	Documents Number	Representation	Citations	SJR *	Cite Score
1	*Resources Policy*	United Kingdom	116	9.2	2912	1.276	6.3
2	*Extractive Industries and Society*	The Netherlands	82	6.5	951	0.999	4.2
3	*Journal of Cleaner Production*	United Kingdom	40	3.2	1384	1.937	13.1
4	*Natural Resources Forum*	United Kingdom	37	2.9	843	0.646	2.9
5	*Science of the Total Environment*	The Netherlands	31	2.5	1492	1.795	10.5
6	*International Journal of Environmental Research and Public Health*	Switzerland	27	2.1	450	0.747	3.4
7	*Minerals and Energy—Raw Materials Report*	United Kingdom	18	1.4	70	0.143	-
8	*Geoforum*	United Kingdom	17	1.4	472	1.584	5.5
9	*World Development*	United Kingdom	17	1.4	820	2.386	8.4
10	*Environmental Research*	United States	16	1.3	677	1.460	7.9

* SJR data was obtained from Scimago Journal & Country Rank.

**Table 3:** Top 10 most cited documents.

Ranking	Authors	Year	Title	Citations	Journal
1	Bebbington et al. [223]	2008	Mining and Social Movements: Struggles Over Livelihood and Rural Territorial Development in the Andes	292	*World Development*
2	Xiao et al. [173]	2017	Soil heavy metal contamination and health risks associated with artisanal gold mining in Tongguan, Shaanxi, China	196	*Ecotoxicology and Environmental Safety*
3	Hilson & Potter [73]	2005	Structural adjustment and subsistence industry: Artisanal gold mining in Ghana	194	*Development and Change*
4	Hylander & Goodsite [157]	2006	Environmental costs of mercury pollution	191	*Science of the Total Environment*
5	Banchirigah [162]	2008	Challenges with eradicating illegal mining in Ghana: A perspective from the grassroots	163	*Resources Policy*
6	Cordy et al. [41]	2011	Mercury contamination from artisanal gold mining in Antioquia, Colombia: The world’s highest per capita mercury pollution	162	*Science of the Total Environment*
7	Fisher [224]	2007	Occupying the margins: Labour integration and social exclusion in artisanal mining in Tanzania	149	*Development and Change*
8	Veiga et al. [218]	2006	Origin and consumption of mercury in small-scale gold mining	149	*Journal of Cleaner Production*
9	Hilson [221]	2009	Small-scale mining, poverty and economic development in sub-Saharan Africa: An overview	141	*Resources Policy*
10	Bose-O’Reilly [167]	2008	Mercury as a serious health hazard for children in gold mining areas	139	*Environmental Research*

**Table 4:** The 15 main words with the highest occurrence in ASM studies.

Ranking	Keywords	Occurrences	Links	Total Link Strength
1	artisanal and small-scale mining	597	88	764
2	mercury	109	41	198
3	mining	80	49	98
4	gold	60	39	129
5	formalization	48	35	101
6	livelihood	38	24	71
7	poverty	36	23	73
8	heavy metals	34	21	53
9	sustainability	25	14	32
10	conflict	23	20	51
11	environment	21	20	52
12	mercury pollution	21	14	30
13	gender	20	19	44
14	sustainable development	20	20	34
15	galamsey	18	19	34

## Data Availability

Not applicable.
